# Neighborhood Ties Reduced Depressive Symptoms in Older Disaster Survivors: Iwanuma Study, a Natural Experiment

**DOI:** 10.3390/ijerph17010337

**Published:** 2020-01-03

**Authors:** Yuri Sasaki, Taishi Tsuji, Shihoko Koyama, Yukako Tani, Tami Saito, Katsunori Kondo, Ichiro Kawachi, Jun Aida

**Affiliations:** 1 Department of International Health and Collaboration, National Institute of Public Health, Wako, Saitama 351-0197, Japan; 2 Department of Social Preventive Medical Sciences, Center for Preventive Medical Sciences, Chiba University, Chiba 263-8522, Japan; tsuji.t@chiba-u.jp (T.T.); kkondo@chiba-u.jp (K.K.); 3 Cancer Control Center, Osaka International Cancer Institute, Osaka 541-8567, Japan; 4 Department of Global Health Promotion, Tokyo Medical and Dental University, Tokyo 113-8510, Japan; yukako.tani@gmail.com; 5 Department of Social Science, Center for Gerontology and Social Science, National Center for Geriatrics and Gerontology, Moriokacho 7-430, Japan; t-saito@ncgg.go.jp; 6 Department of Gerontological Evaluation, Center for Gerontology and Social Science, National Center for Geriatrics and Gerontology, Moriokacho 7-430, Japan; 7 Center for Well-Being and Society, Nihon Fukushi University, Aichi 470-3295, Japan; 8 Department of Social and Behavioral Sciences, Harvard School of Public Health, Boston, MA 02115, USA; ikawachi@hsph.harvard.edu; 9 Department of International and Community Oral Health, Tohoku University Graduate School of Dentistry, Sendai, Miyagi 980-8577, Japan; aidajun@m.tohoku.ac.jp

**Keywords:** neighborhood ties, depressive symptoms, natural disaster, older survivors, social capital, resilience

## Abstract

*Objective*: As most studies relating to mental health and disasters have employed cross-sectional or follow-up assessments about psychological health with post-disaster information, the association between changes in social ties and mental health remains unclear. We examined the relationship between the changes in survivor neighborhood ties and depressive symptoms before and after a natural disaster. *Methods*: Participants were 3567 individuals aged ≥65 years living in Iwanuma city who had responded to questionnaires by the Japan Gerontological Evaluation Study both predating the 2011 Great East Japan Earthquake and Tsunami, and 2.5 years afterward. Changes in the depressive symptoms were assessed using the geriatric depression scale (GDS) at the baseline and follow-up survey. Changes in the neighborhood ties were assessed by asking the participants about their interactions with people in their neighborhood. Possible confounders were adjusted in a linear regression model. *Results*: Among the 3111 participants in this analysis, 1073 (34.5%) had increased GDS score after the disaster. There were 336 (10.8%) individuals who had neighborhood ties before the disaster, but had no ties afterward; their mean GDS score increased from 2.93 points in 2010 to 3.19 points in 2013. Among those who had not had ties before and after the disaster the mean GDS score remained almost stable, from 2.19 points in 2010 to 2.12 points in 2013. The participants with post-disaster ties were significantly less likely to have an increased GDS score compared with those who had not had ties before and after the disaster (β = −0.39; 95% confidence interval: −0.72, −0.06). *Conclusions*: Increased neighborhood ties after the disaster reduced the risk of depressive symptoms even when survivors suffered disaster damages. The study reinforces the importance of social capital in disaster recovery and suggests to local governments and local communities that fostering horizontal, neighborhood ties may improve disaster preparedness and mental health resilience.

## 1. Introduction

The number of natural disasters worldwide has been increasing [1], and they have wide-ranging psychological impacts. A systematic review and meta-analysis suggest that psychological distress and psychiatric disorder increase after natural disasters [2]. The proportion of people who suffer from a post-traumatic stress disorder and depressive symptoms increases after a disaster [3,4,5,6]. In relation to the recovery of psychiatric disorders after the Great East Japan Earthquake in 2011, a systematic review reported a long-term improvement in the posttraumatic stress reaction, however, concerning depression, no study showed its decline even two years after the disaster’s occurrence [7]. Consequently, approaches to reduce the burden of depression after a natural disaster is a significant concern.

Social tie/support is a known protective factor against the negative psychological impact of natural disasters. Previous studies have consistently suggested that the social tie/support buffers depression and other psychological problems [8,9,10,11,12,13]. Social ties refer to the connections to and contacts with other people through membership. They include not only family members, relatives, and friends (primary groups), but also those who are less personal; work, voluntary, and religious organizations are examples (secondary groups) [14].

As most studies relating to mental health and disaster have employed cross-sectional or follow-up assessments about psychological health with post-disaster information [15,16,17], the association between the changes in the ties and psychological health using pre- and post-disaster information is unclear. Additionally, the association between social ties and psychological health has not been investigated focusing on older adults who form an increasing proportion of those exposed to natural disasters in this aging world [18,19]. Understanding the possible influence of the changes in the social ties during natural disasters on the psychological health among older survivors can potentially assist policymakers to prepare for future disasters.

On 11 March 2011, a magnitude-9 earthquake and tsunami struck northeast Japan. The disaster killed more than 15,000 people and more than 2500 people are still missing [20]. Japan is prone to earthquakes. In the Tokai region as well as in the Pacific to the southeast and south of Japan, major earthquakes that directly hitting the Tokyo area are predicted in the near future [21]. 

Under these circumstances, physical infrastructures such as buildings, seismic standards, and breakwaters are important to reduce disaster damage. However, the limited capacity of physical infrastructures has been recognized. During the Great East Japan Earthquake, the tsunami destroyed the breakwater in Kamaishi-city, the deepest breakwater in the world at that moment which was certified by the Guinness Book of Records. In recent years, it has been pointed out that attention should be paid to social infrastructures although large efforts have been aimed at physical infrastructures [22,23].

We focused on the social ties with the neighborhood, because a person’s social tie influences the receipt of various kinds of social support [14,24,25], and people with a higher level of neighborhood belonging try to maintain their connection to other people by checking if they are all right during a disaster and are willing to offer help to them if possible and if necessary [26]. The neighborhood can be either a primary or a secondary group of social ties; thus, it has the potential to influence the psychological health of older survivors. In addition, previous studies reported that social capital, resources obtained from social ties and it has three aspects of bonding, bridging, and linking [27,28], played a critical role in communities [29]. However, fewer quantitative studies examined their impacts on disaster preparedness and resilience.

A natural experiment is an observational study in which an event or a situation that allows for the random or seemingly random assignment of study participants to different groups to answer a particular question [30]. Taking advantage of a unique ‘natural experiment’, we used information about the psychological health status as well as existing neighborhood ties of community-dwelling older adults seven months before the 2011 Great East Japan Earthquake and Tsunami. Utilizing this design, we examined the relationship between the changes in the social ties with neighbors and depressive symptoms in older adults who survived an earthquake disaster. We hypothesized that the risk of depressive symptoms in the older survivors would be smaller among those who increased their social ties after the disaster. 

## 2. Materials and Methods

### 2.1. Study Design and Participants

This study was part of the Japan Gerontological Evaluation Study (JAGES), which began in 2010 as a nationwide, population-based, prospective cohort study investigating the predictors of physical and psychological health in community-dwelling Japanese older adults [31,32,33]. In the present longitudinal study, we used the panel data from two waves of the JAGES survey.

**Figure 1:**
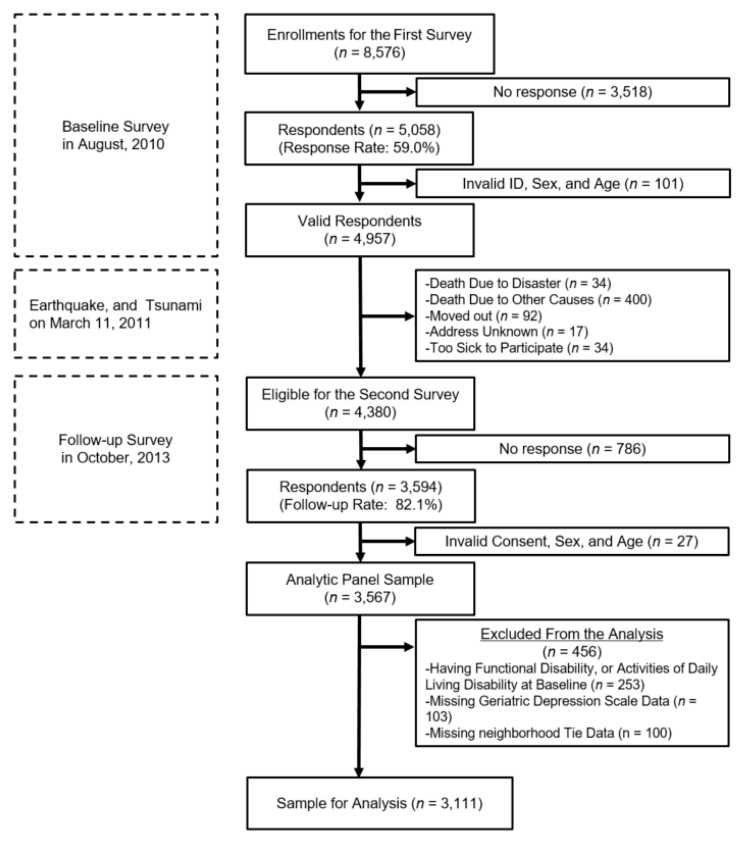
Participant flow of the present study.

One of the original field sites of the JAGES cohort was Iwanuma city, Miyagi prefecture, which is located roughly 80 km west of the epicenter of the 2011 earthquake. Surveys were mailed to all residents of this city aged 65 years or older in August 2010 (that is, seven months before the disaster) and again after the disaster, in October 2013. The response rate to the baseline survey was 59.0% (*n* = 5058). Of these, 34 people lost their lives on the day of the disaster, and an additional 400 people died of natural causes before the follow-up survey. After excluding people who moved out of the area (n = 92), were lost to follow-up with no known forwarding address (*n* = 17), or were too sick to be re-contacted (*n* = 34), 4380 people were eligible for the second survey. Among them, 3594 people responded to the second survey (response rate of 82.1%). After excluding invalid consent forms, 3567 people participated in both surveys in 2010 and 2013, and 3111 people were included in the analysis (response rate when those excluded from the analysis were taken into account: 71.0%; Figure 1. Participant flow of the present study) [31].

We used the data from 3111 participants (1417 males and 1694 females), excluding those with missing data on the 15-item Geriatric Depression Scale (GDS) and neighborhood ties, participants who reported limitations in activities of daily living at baseline, such as dependence in walking, bathing, and toileting, and those who received public long-term care insurance benefits. Further details of the Iwanuma study have been previously described [34,35,36].

### 2.2. Outcome Variable: Geriatric Depression Scale

In both the 2010 and 2013 surveys, we assessed the depressive symptoms measured by the Japanese short version of the GDS (GDS-15), which has been previously validated [37]. The validity and reliability of the GDS, including this 15-item version, for assessing depressive symptoms in older populations has long been established [37,38]. The GDS consists of a simple yes/no format that is suitable for self-administration [38,39]. Following the previous research, we imputed the overall score based on the average of the available items for handling the missing values [34,36]. The outcome variable in the present study was the difference in the GDS score between baseline (2010) and follow-up (2013), calculated by subtracting the 2010 GDS score from the 2013 score. If the value for a participant was positive, the depressive symptoms of that person in 2013 had worsened from 2010 [34].

### 2.3. Predictor Variable: Change in the Social Ties with Neighbors before and after the Disaster

Neighborhood ties before and after the disaster in 2011 were assessed by asking, “What kind of interactions do you have with people in your neighborhood?” Possible responses were (1) mutual consultation, lending and borrowing daily commodities, and cooperation in daily life; (2) standing and chatting frequently; (3) no more than exchanging greetings; or (4) none, not even greetings. The responses were categorized as ‘having ties (combining responses 1 and 2)’ or ‘having no tie (combining responses 3 and 4)’. We compared the individuals who kept no ties before and after the disaster, with the others (those who maintained the ties, those who increased the ties, and those who decreased the tie after the disaster).

## 3. Covariates: Sociodemographic Characteristics and Disaster Damage

The demographic characteristics before the disaster in 2010 (sex and age), the changes in the survivors’ characteristics before and after the disaster (medical treatment, living status, physical activity, smoking behavior, drinking behavior, employment status, self-rated economic situation, and walking behavior), and disaster damages (relocation, and losing close relative(s) and friend(s) in the disaster) were adjusted in a linear regression model.

Information on sex and age was obtained from the government register in 2010. Information on the educational level (<6 years, 6–9,10–12, or =>13), medical treatment (receiving treatment or not), living status (living alone or not), physical activity (frequency of sports club/group participation), smoking behavior (present smoker or nonsmoker), drinking behavior (present drinker or nondrinker), employment status (working or not), self-rated economic situation (having economic difficulty or not), walking per day (<30 min or =>30 min), were obtained from the self-reported questionnaires from the baseline and follow-up surveys. Loss of relationships due to the disaster was evaluated via a question ‘Did you lose a close relative(s) or friend(s) in the earthquake?’ for which participants could provide multiple possible answers. Their answers were categorized as follows: losing close relative(s) or not, and losing close friend(s) or not. 

Each participant was also asked about his/her experience of relocation after the disaster. The responses were combined into three categories, as previously reported [40]: (1) no relocation; (2) group relocation into a prefabricated housing; (3) individual relocation into a prefabricated housing; (4) an existing private accommodation; or (5) newly established housing. The variance inflation factor (VIF) was measured to analyze the magnitude of the multicollinearity of model terms (Appendix A). A variable that showed the VIF more than five with other variables was excluded from the final model.

### 3.1. Statistical Analysis

Among the analytical subjects of 3111, we calculated the rates of each category for socio-demographic variables. Paired *t*-tests were used to compare GDS scores in different social ties groups. We also used a linear regression model to examine the association between the change in the neighborhood ties and the change in the GDS scores [34]. Skewness and kurtosis were calculated for the distribution of GDS scores in 2010 and 2013. The GDS score changes were entered as continuous variables. The multivariate adjusted results were expressed as non-standardized coefficients with 95% confidence intervals (CI). We used STATA14 (StataCorp, College Station, TX, USA) for all statistical analyses with the statistical significance level set at *p* < 0.05.

### 3.2. Ethical Considerations

The survey protocol was approved by the human subjects’ committee of the Harvard T.H. Chan School of Public Health (CR-23143) as well as the human subjects’ committees of Tohoku University (21-40, 24-29), Nihon Fukushi University (10-05, 13-14), and Chiba University (2493). Informed consent was obtained at the time of data collection. Voluntary participation and right of withdrawal at any time were assured. This study conformed to the principles embodied in the Declaration of Helsinki.

## 4. Results

The average age of the participants at baseline was 73 ± 5.9 years and 54.5% of them were women (Table 1). Among the 3111 participants, 1073 (34.5%) increased the GDS score after the disaster in 2013. Number of individuals who increased neighborhood ties after the disaster was 257 (8.3%).

**Figure 2:**
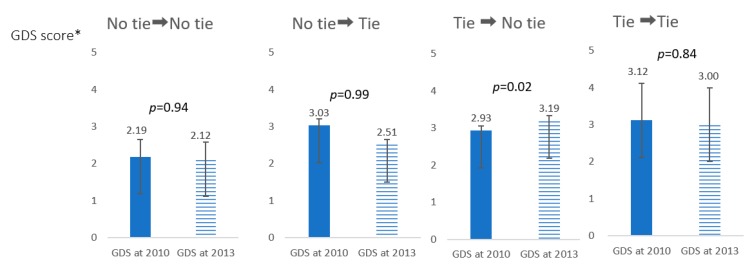
Change of Mean Geriatric Depression Scale (GDS) score from 2010 to 2013. * GDS score is up to 15 scores. The higher the score, the more depressed. Means of GDS and standard deviations are presented.

Among the individuals who had ties before the disaster, but had no ties after the disaster, mean GDS score increased from 2.93 points at 2010 to 3.19 points at 2013, although it was almost stable from 2.19 points at 2010 to 2.12 points at 2013 among those who had not had ties before and after the disaster (Figure 2).

Table 2 shows the multivariate adjusted association between the changes in the neighborhood ties and the changes in the GDS scores. The individuals who had no neighborhood ties before the disaster, but had ties afterward were significantly less likely to have an increased GDS score compared with those who had no ties before and after the disaster (β = −0.39, 95% CI: −0.72, −0.06).

Compared with the individuals who evaluated their economic situation as difficult before and after the disaster, those who evaluated it as not difficult before the disaster, but difficult afterward, were significantly more likely to have worsened GDS scores (β = 0.68, 95% CI: 0.14, 1.22). Contrary, the individuals who evaluated it as difficult before the disaster, but not difficult afterward, were significantly less likely to have worsened GDS scores (β = −0.57, 95% CI: −1.08, −0.07).

In addition, the changes in the life style behaviors were significantly associated with the changes in the GDS score. Both individuals who stopped smoking, and who started smoking after the disaster, were more likely to have worsened GDS scores compared with those who kept smoking after the disaster (β = 1.05, 95% CI: 0.44, 1.66; β = 1.35, 95% CI: −0.23, 2.92, respectively). Individuals who stopped drinking alcohol after the disaster were also more likely to have worsened GDS scores compared with those who kept drinking (β = 0.36, 95% CI: 0.01, 0.71). Individuals who walked 30 min/day or more before the disaster, but walked less than 30 min/day afterward were significantly more likely to have worsened GDS scores than those who walked less than 30 min/day before and after the disaster (β = 0.51, 95% CI: 0.15, 0.86).

## 5. Discussion

The main contribution of this study is that we have analyzed whether the changes in the neighborhood ties before and after a natural disaster function as an indicator of depressive symptom prevention in older survivors. Overall GDS scores were low and in the normal range. However, a total of 34.5% of the older survivors had worsened GDS scores after the disaster (Table 1). In line with the hypothesis, the survivors who had no neighborhood ties before the disaster, but had ties afterward, had a lower risk of increasing GDS scores than that of those who had not have the ties before and after the disaster (Table 2). We also found that formatting ties were protective, but maintaining the status quo did not seem to increase the risk of developing depression (Figure 2).

The results of our present study are consistent with those of previous studies about the positive influence of neighborhood ties on psychological health after natural disasters [8,9,10,11,12,13]. However, to the best of our knowledge, no studies have focused on the influence of the changes in the neighborhood ties before and after a disaster among older adults. Even the survivors who originally did not have neighborhood ties must have had a need to cooperate with their surroundings after the disaster. In these survivors, the worsening of the depressive symptoms may have been prevented.

However, survivors who needed to get in touch with their neighbors might have been heavily damaged by the disaster. Therefore, the variables regarding the disaster damages were also adjusted for. Even after adjusting for such variables, the influence of the neighborhood ties remained significant.

The influence of the time passed since the earthquake may have also influenced the results of this study. Watanabe indicated that the benefits of social support vary across time and according to the source of support. In the short term (6-month post-earthquake), lower levels of depressive symptoms among older survivors were associated with higher levels of child and extended family support. In contrast, in the longer term (12-month post-earthquake), lower levels of depressive symptoms were associated with higher levels of support by extended family and neighbors. Thus, the social support by extended family and neighbors would play a more important role in promoting psychological health in the later phases of recovery [10]. These supports promote positive thinking and self-esteem in survivors and they can eventually attain better psychological status and well-being [10,41]. Since our follow-up survey was conducted approximately 2.5 years after the disaster, there may have been a large influence by the neighbors.

Cultural differences in seeking and using neighborhood ties should also be considered. A study on culture and social support found that Asians and Asian Americans benefit psychologically to a greater extent from implicit social support (“focusing on valued social groups”) than from explicit social support (“seeking and using advice and emotional solace”); the reverse was true for European Americans [42]. That is, the influence of neighborhood ties may vary depending on the cultural emphasis on maintaining harmonious social relationships on the one hand, versus an emphasis on self-expression and verbal sharing of thoughts and feelings (as in western settings) [42,43].

The implication of this study is that fostering neighborhood ties after a natural disaster may be able to alleviate the deterioration of depression, even among the older survivors who did not have neighborhood ties before the disaster; in other words, social ties with neighbors might function as a resource for the survivors’ psychological health even in disaster affected areas. Public health interventions can also increase older people’s opportunities to participate in social activities and improve their social interactions after the disaster via physical activities such as walking exercise, which had a significant association with the changes in the depressive symptoms both in this study and a previous one [44].

Our results should be interpreted with some caution. Since we assessed the presence of depressive symptoms via the participants’ recall, the results do not necessarily translate to clinical significance. However, mitigating impact on the depressive symptom worsening due to the increasing of neighborhood ties after the disaster (β= −0.39) was more than half of the negative impact of economic difficulty after the disaster (β = 0.68), and comparable with the worsening impact of changing drinking behaviors after the disaster (β = 0.36) (Table 2).

Our study limitations also included numbers of missing information for some study characteristics (Table 1), which could produce information bias. In addition, due to the nature of earthquake disasters, the sample was naturally restricted regarding geography and culture. Even without a disaster occurring, residential areas have been found to be associated with depression in Japanese older adults [45]. Therefore, the changes in the depressive symptoms may overestimate the disaster impact, since there was a 7-month time lag between the assessment of the depressive symptoms and the exposure to the disaster. We also cannot deny the possibility of reverse causality because we verified the changes of two variables at two time points.

Moreover, future research is needed to explore not only the changes in the neighborhood ties, but also the types of ties. For example, there are bonding ties that connect similar people as well as bridging social ties that bring people from diverse groups together (e.g., race, class, age, etc.).

The present study also had several strengths. Using the pre-disaster data, we were able to determine the influence of the change of disaster preparedness resources. In addition, because of Japan’s compulsory system of domiciliary registration, which requires all residents to notify authorities of address changes, the number of individuals who dropped out at follow-up in our dataset was quite low (11.6%); consequently, the degree of bias induced by loss to follow-up was likely small [34].

## 6. Conclusions

Older survivors with increased neighborhood ties after a major natural disaster had a lower risk of depressive symptoms worsening than those who had not have before and after the disaster, regardless of the damages they suffered. As natural disasters become more frequent, it will become even more important to monitor the neighborhood ties of survivors and their psychological health in vulnerable populations, including older individuals. The long-term beneficial effects of neighborhood ties on disaster survivor resilience mental should be considered in strategic planning to mitigate future disasters, especially in countries where natural disasters are a cyclic occurrence. Future research should explore the specifics of community ties (e.g., bonding, bridging, linking) to inform better metrics in policy recommendations.

## Figures and Tables

**Table 1:** Socio-demographic characteristics (*n* = 3111).

		*n*		%
GDS scores at 2010	Mean ± SD	2.4	±	2.3
GDS scores at 2013	Mean ± SD	2.4	±	2.4
Change of GDS scores (2010→2013)	Increase (get worse)	1073		34.5
	No change	840		27.0
	Decrease	1198		38.5
GDS score at 2010	GDS score <5	2563		82.4
	GDS score 5–9	534		17.2
	GDS score ≥10	14		0.5
GDS score at 2013	GDS score <5	2572		82.7
	GDS score 5–9	520		16.7
	GDS score ≥10	19		0.6
Change in neighborhood tie (2010→2013)	No tie➡No tie	2195		70.6
	No tie➡Tie	257		8.3
	Tie➡No tie	336		10.8
	Tie➡Tie	323		10.4
Sex	Male	1417		45.6
	Female	1694		54.5
Age	Mean ± SD	73	±	5.9
Educational level	<6	28		0.9
	6–9	999		32.1
	10–12	1351		43.4
	=>13	659		21.2
	Missing	74		2.4
Change in receiving medical treatment (2010→2013)	Treat➡Treat	2284		73.4
	Treat➡Non	98		3.2
	Non➡Treat	392		12.6
	Non➡Non	295		9.5
	Missing	42		1.4
Change in living status (2010→2013)	Alone➡Alone	239		7.7
	Alone➡Not alone	21		0.7
	Not alone➡Alone	89		2.9
	Not alone➡Not alone	2648		85.1
	Missing	114		3.7
Change in equivalized income (2010→2013)	No change	568		18.3
	Increase	791		25.4
	Decrease	969		31.2
	Missing	783		25.2
Change in sports club participation (2010→2013)	No change	1828		58.8
	Increase	367		11.8
	Decrease	389		12.5
	Missing	527		16.9
Change in smoking behavior (2010→2013)	Smoke➡Smoke	232		7.5
	Smoke➡Non	105		3.4
	Non➡Smoke	17		0.6
	Non➡Non	2533		81.4
	Missing	224		7.2
Change in drinking behavior (2010→2013)	Drink➡Drink	971		31.2
	Drink➡Non	216		6.9
	Non➡Drink	66		2.1
	Non➡Non	1797		57.8
	Missing	61		2.0
Change in employment status (2010→2013)	No work➡No work	2151		69.1
	No work➡Work	78		2.5
	Work➡No work	207		6.7
	Work➡Work	311		10.0
	Missing	364		11.7
Change in self-rated economic situation (2010→2013)	Difficulty➡Difficulty	193		6.2
	Difficulty➡No difficulty	314		10.1
	No difficulty➡Difficulty	177		5.7
	No difficulty➡No difficulty	2248		72.3
	Missing	179		5.8
Change in walking/day behavior (2010→2013)	Walk < 30 min➡Walk < 30 min	605		19.5
	Walk < 30 min➡Walk => 30 min	448		14.4
	Walk => 30 min➡Walk < 30 min	374		12.0
	Walk => 30 min➡Walk => 30 min	1560		50.1
	Missing	124		4.0
Housing damage	Yes	1793		57.6
	No	1247		40.1
	Missing	71		2.3
Lost family members/relatives	Yes	830		26.7
	No	2281		73.3
Lost friends	Yes	488		15.7
	No	2623		84.3
Relocation after the disaster	No relocation	2872		92.3
	Group relocation to prefabricated housing	63		2.0
	Individual relocation to prefabricated housing	7		0.2
	Existing private accommodation	28		0.9
	Newly established housing	48		1.5
	Missing	93		3.0

GDS: Geriatric Depression Scale; SD: Standard Deviation.

**Table 2:** Multivariate adjusted association of the change in the neighborhood ties with the change in the GDS score among the survivors from the 2011 disaster in Japan.

Variable		B	*β*	SE	95% CI		*p* Value
Change in neighborhood tie	No tie➡No tie						
(2010→2013)	No tie➡Tie	−0.05	−0.39	0.17	−0.72	−0.06	0.02
	Tie➡No tie	0.02	0.15	0.15	−0.15	0.45	0.32
	Tie➡Tie	0.00	0.01	0.15	−0.28	0.30	0.95
Sex	Male						
	Female	−0.03	−0.12	0.12	−0.35	0.11	0.32
Age		0.02	0.01	0.01	−0.01	0.02	0.46
Change in receiving medical treatment	Treat➡Treat						
(2010→2013)	Treat➡Non	−0.02	−0.18	0.23	−0.63	0.28	0.45
	Non➡Treat	0.02	0.14	0.13	−0.11	0.39	0.27
	Non➡Non	−0.01	−0.04	0.13	−0.29	0.22	0.79
Change in living status	Alone➡Alone						
(2010→2013)	Alone➡Not alone	−0.02	−0.58	0.65	−1.86	0.70	0.38
	Not alone➡Alone	0.01	0.12	0.33	−0.52	0.76	0.72
	Not alone➡Not alone	−0.04	−0.24	0.17	−0.57	0.10	0.17
Change in sports club participation	No change						
(2010→2013)	Increase	−0.02	−0.13	0.12	−0.36	0.10	0.26
	Decrease	0.02	0.11	0.12	−0.12	0.35	0.34
Change in smoking behavior	Smoke➡Smoke						
(2010→2013)	Smoke➡Non	0.10	1.05	0.31	0.44	1.66	0.00
	Non➡Smoke	0.04	1.35	0.80	−0.23	2.92	0.09
	Non➡Non	0.02	0.13	0.17	−0.20	0.46	0.44
Change in drinking behavior	Drink➡Drink						
(2010→2013)	Drink➡Non	0.05	0.36	0.18	0.01	0.71	0.04
	Non➡Drink	−0.03	−0.36	0.29	−0.92	0.20	0.21
	Non➡Non	0.02	0.09	0.11	−0.14	0.31	0.45
Change in self-rated economic situation	Economic difficulty➡Economic difficulty						
(2010→2013)							
	Economic difficulty➡No economic difficulty	−0.09	−0.57	0.26	−1.08	−0.07	0.03
	No economic difficulty➡Economic difficulty	0.08	0.68	0.28	0.14	1.22	0.01
	No economic difficulty➡No economic difficulty	0.02	0.12	0.22	−0.31	0.54	0.59
Change in walking/day behavior	Walk < 30 ➡Walk < 30						
(2010→2013)	Walk < 30 ➡Walk => 30	−0.02	−0.09	0.17	−0.42	0.25	0.60
	Walk => 30➡Walk < 30	0.08	0.51	0.18	0.15	0.86	0.01
	Walk => 30➡Walk => 30	0.02	0.07	0.12	−0.17	0.31	0.58
Lost family members/relatives	Lost						
	No lost	−0.04	−0.17	0.10	−0.37	0.02	0.08
Lost friends	Lost						
	No lost	0.03	0.18	0.12	−0.07	0.42	0.16
Relocation after the disaster	No replacement						
	Group relocation to prefabricated housing	0.04	0.72	0.41	−0.08	1.52	0.08
	Individual relocation to prefabricated housing	−0.02	−0.58	0.71	−1.97	0.81	0.41
	Existing private accommodation	0.03	0.67	0.48	−0.27	1.61	0.16
	Newly established housing	−0.01	−0.14	0.34	−0.80	0.52	0.67
					R-squared = 0.053		

CI: Confidence Interval; GDS: Geriatric Depression Scale; SE: Standard Error.

## Appendix A

**Table A1:** Measurement of Variance Inflation Factor (VIF).

	Variable	VIF	1/VIF
Change in neighborhood tie (2010→2013)	No tie➡No tie		
	No tie➡Tie	1.04	0.96
	Tie➡No tie	1.09	0.92
	Tie➡Tie	1.09	0.92
Sex	Female	1.63	0.61
Age	Age	1.16	0.86
Change in receiving medical treatment (2010→2013)	Treat➡Treat		
	Treat➡Non	1.03	0.97
	Non➡Treat	1.06	0.94
	Non➡Non	1.06	0.94
Change in living status	Alone➡Alone		
(2010→2013)	Alone➡Not alone	1.10	0.91
	Not alone➡Alone	1.36	0.74
	Not alone➡Not alone	1.48	0.68
Change in sports club participation (2010→2013)	No change		
	Increase	1.07	0.93
	Decrease	1.06	0.94
Change in smoking behavior (2010→2013)	Smoke➡Smoke		
	Smoke➡Non	1.44	0.69
	Non➡Smoke	1.07	0.93
	Non➡Non	1.61	0.62
Change in drinking behavior (2010→2013)	Drink➡Drink		
	Drink➡Non	1.18	0.84
	Non➡Drink	1.06	0.95
	Non➡Non	1.73	0.58
Change in self-rated economic situation(2010→2013)	Economic difficulty➡Economic difficulty		
	Economic difficulty➡No economic difficulty	2.51	0.40
	No economic difficulty➡Economic difficulty	1.86	0.54
	No economic difficulty➡No economic difficulty	3.15	0.32
Change in walking/day behavior (2010→2013)	Walk < 30➡Walk < 30		
	Walk < 30➡Walk => 30	1.54	0.65
	Walk => 30➡Walk < 30	1.44	0.69
	Walk => 30➡Walk => 30	1.90	0.53
Lost family members/relatives	Lost		
	No lost	1.06	0.94
Lost friends	Lost		
	No lost	1.05	0.95
Relocation after the disaster	No replacement		
	Group relocation to prefabricated housing	1.04	0.96
	Individual relocation to prefabricated housing	1.01	0.99
	Existing private accommodation	1.06	0.94
	Newly established housing	1.04	0.97
	Mean VIF	1.35	
